# Association between maternal triglycerides and disturbed glucose metabolism in pregnancy

**DOI:** 10.1007/s00592-020-01644-z

**Published:** 2021-01-02

**Authors:** Daniel Eppel, Michael Feichtinger, Tina Lindner, Grammata Kotzaeridi, Ingo Rosicky, Guelen Yerlikaya-Schatten, Wolfgang Eppel, Peter Husslein, Andrea Tura, Christian S. Göbl

**Affiliations:** 1 Department of Obstetrics and Gynecology, Division of Obstetrics and Feto-Maternal Medicine, Medical University of Vienna, Vienna, Austria; 2 Wunschbaby Institut Feichtinger, WIF, Vienna, Austria; 3 Metabolic Unit, CNR Institute of Neuroscience, Padova, Italy

**Keywords:** Hyperlipidemia, Insulin resistance, Pregnancy, *β*-cell function

## Abstract

**Aims:**

Dyslipidemia in pregnancy is associated with adverse pregnancy outcomes as elevated triglycerides might be considered as a risk factor for hyperglycemia and gestational diabetes. As only a few studies have addressed the association between maternal triglycerides and glucose metabolism, we aimed to explore the pathophysiologic associations of moderate hypertriglyceridemia and maternal glucose metabolism in pregnancy.

**Methods:**

Sixty-seven pregnant women received a detailed metabolic characterization at 12+0–22+6 weeks of gestation by an extended 2h-75g OGTT (oral glucose tolerance test); with measurements of glucose, insulin and C-peptide at fasting and every 30 min after ingestion and assessment of triglycerides at fasting state. All examinations were repeated at 24+0–27+6 weeks of gestation.

**Results:**

Elevated triglycerides in early gestation were associated with insulin resistance and β-cell dysfunction. Mean glucose concentrations during the OGTT in early pregnancy were already higher in women with hypertriglyceridemia as compared to women with triglycerides in the normal range. A higher degree of insulin resistance and increased OGTT glucose levels were also observed when metabolic assessments were repeated between 24 and 28 weeks of gestation. Of note, elevated triglycerides at early gestation were associated with development of gestational diabetes by logistic regression (odds ratio: 1.16, 95%CI: 1.03–1.34, *p*=0.022 for an increase of 10 mg/dl).

**Conclusions:**

Hypertriglyceridemia at the start of pregnancy is closely related to impaired insulin action and *β*-cell function. Women with hypertriglyceridemia have higher mean glucose levels in early- and mid-gestation. Pregnant women with elevated triglycerides in early pregnancy are at increased risk of developing gestational diabetes.

**Electronic supplementary material:**

The online version of this article (10.1007/s00592-020-01644-z) contains supplementary material, which is available to authorised users.

## Background

Gestational diabetes mellitus (GDM) is widely accepted as a major reason for adverse pregnancy outcomes including fetal macrosomia, cesarean section rate, neonatal hypoglycemia or the development of obesity in the newborns’ later life [1–3]. In addition to the well-known effect of hyperglycemia, other metabolic characteristics could be of further importance for maternal and offspring health [4, 5]. For example, previous studies indicated that alterations in maternal lipid metabolism were associated with adverse pregnancy outcomes, as elevated serum triglycerides concentrations were found to be associated with an increased risk of preeclampsia and the development of large for gestational age neonates [6]. As known from studies of non-pregnant women, maternal lipids may be intercorrelated with obesity and parameters of disturbed glucose metabolism (especially insulin resistance), which are commonly known risk factors for the development of hyperglycemia [7]. Therefore, increased maternal lipid content could be regarded as a potential risk factor for hyperglycemia in pregnancy as well [4]. However, only a few studies have addressed the possible cross-link between maternal triglyceride concentrations and parameters of glucose metabolism during gestation, and consequently, the evidence for possible clinical implications of increased lipid concentrations in pregnancy is sparse.

Therefore, this study aims to assess the pathophysiologic characteristics of subclinical hyperlipidemia in early pregnancy. In particular, associations between maternal triglycerides and parameters of glucose metabolism (including insulin sensitivity, insulin secretion and *β*-cell function) are described, and their possible association with the consecutive development of GDM is assessed as a secondary objective.

## Participants and methods

### Study design and participants

The present explorative study aimed at identifying risk factors for glucometabolic disorders in pregnancy as published in detail elsewhere [8]. In short, sixty-seven pregnant women received a detailed metabolic characterization at the first study visit (median gestational age: 18.3 weeks, IQR: 16.0–20.3 weeks) by an extended 2h-75g OGTT (with measurements of glucose, insulin and C-peptide at fasting and every 30 min after ingestion), and laboratory assessment of triglycerides (TG) at fasting state, as well as anthropometric measurements. For descriptive purposes, the study participants were classified according to their triglycerides concentration in early gestation: normal triglycerides (NTG) if TG was equal or below 150 mg/dl and moderate hypertriglyceridemia (HTG) if TG exceeded 150 mg/dl, according to several guidelines [9, 10]. All examinations were repeated at 24+0–27+6 weeks of gestation. GDM was diagnosed by using the IADPSG criteria [11]. Patients with pre-existing diabetes were not included in this study. All laboratory parameters were measured according to the standard laboratory methods at our certified Department of Medical and Chemical Laboratory Diagnostics (http://www.kimcl.at). The study was approved by the Ethics Committee of the Medical University of Vienna and performed in accordance with the Declaration of Helsinki. All participants gave written informed consent.

### Calculation of parameters of glucose homeostasis

The quantitative insulin sensitivity check index (QUICKI) was used to assess insulin sensitivity at fasting state [12]. Moreover, we used dynamic indices of insulin action from OGTT data such as the composite index (ISI-comp) as well as the most recently developed PREDIM index to quantify whole- body insulin action [13, 14]. Overall insulin response to glucose was calculated by use of a modified insulinogenic index (AUC-insulin/AUC-glucose 0–120) [15]. The extent to which the pancreatic *β*-cells can adapt to impaired insulin sensitivity was examined by the ISSI-2 index (sometimes called the oral disposition index) as ISI-comp × AUC-insulin/AUC-glucose 0–120. In addition, basal insulin secretion (BIS) and total insulin secretion rate (TIS) were assessed by mathematical modelling from insulin and C-peptide data according to [16].

### Statistical analysis

Categorical variables were summarized by counts and percentages and compared by Fisher’s exact test. Continuous scaled variables were summarized by means and standard deviations and compared by Student’s t test. Skewed distributed parameters were summarized by mean and interquartile range and compared by an exact Wilcoxon rank-sum test. Analysis of covariance (ANCOVA) was used to adjust for possible confounders like differences in maternal BMI (body mass index). Bivariate associations between ordinal and metric scaled variables were assessed by Spearman’s correlation coefficient.

Statistical analysis was performed by R (V 3.5.3) and contributing packages. The two-sided significance level was set to 0.05. However, p-values were interpreted in an explorative manner, and therefore, there was no further adjustment for multiplicity if not otherwise indicated in the manuscript.

## Results

**Figure 1:**
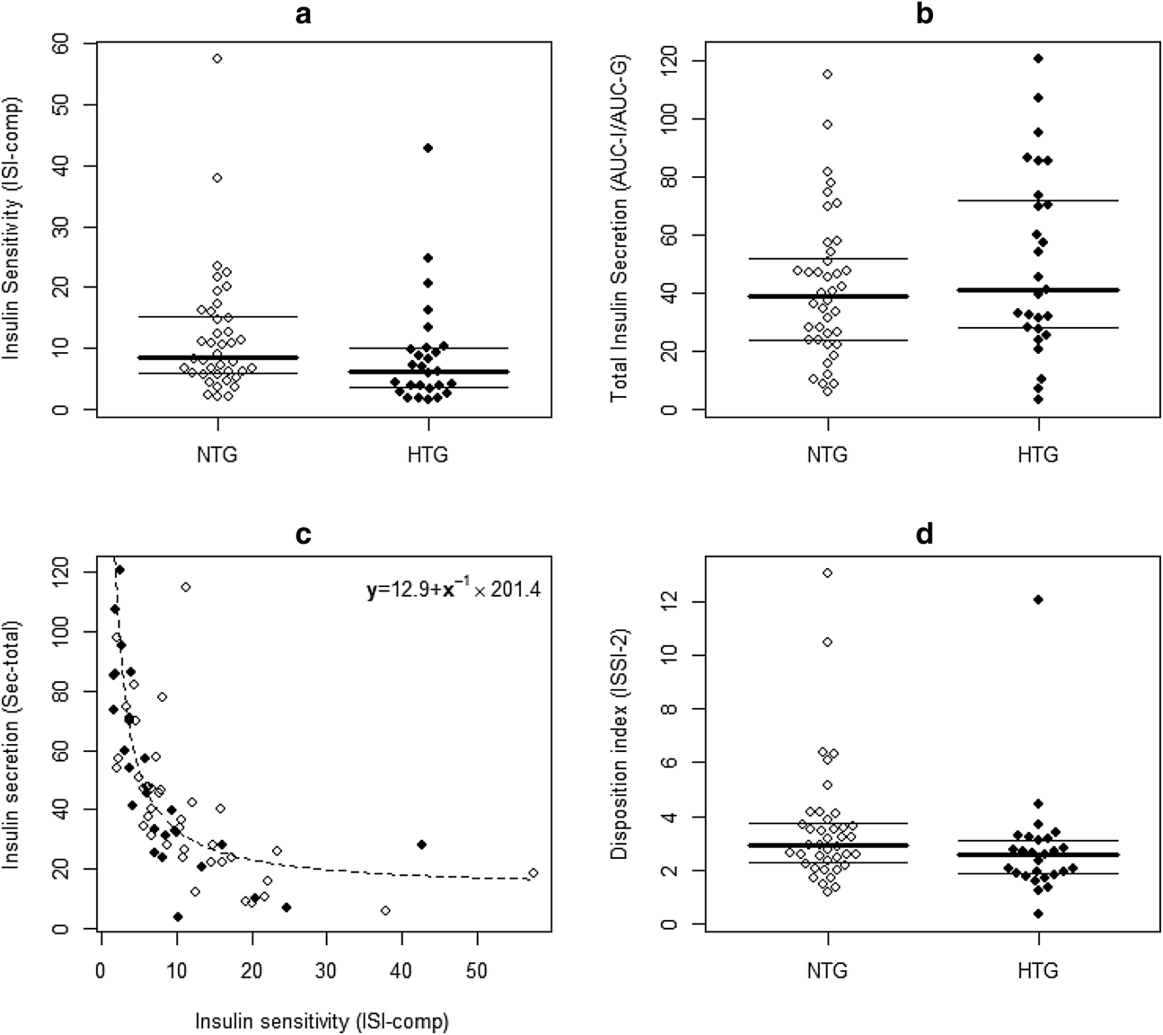
Bee swarm plots representing comparisons of insulin action (**a**) and insulin secretion (**b**), the association between insulin sensitivity and secretion showing the hyperbolic regression line (**c**) and the comparison of the oral disposition index (**d**) at early gestation. NTG, normal triglycerides; HTG, hypertriglyceridemia.

Twenty-seven patients with moderate hypertriglyceridemia were identified. As shown in Table 1 elevated triglycerides in early gestation were associated with impaired insulin sensitivity as expressed by significantly lower index levels of PREDIM as well as higher BMI, and the differences in insulin action between the subgroups remained significant in multivariable analysis after adjustment for pregestational BMI and age (adjusted mean difference for PREDIM after log transformation: 0.15, 95%CI: 0.02–0.29, *p*=0.028). The higher insulin resistance observed in women with hypertriglyceridemia was accompanied by higher basal and total insulin secretion, although lower ISSI-2 index indicated insufficient compensation of insulin secretion for the observed degree of insulin resistance (Fig. 1). As a consequence, already at the beginning of pregnancy, mean glucose concentrations during the OGTT were increased in women with moderate hypertriglyceridemia, as compared to women with triglycerides in the normal range. Higher degrees of insulin resistance and increased OGTT glucose levels were also observed when metabolic assessments were repeated between 24 and 28 weeks of gestation (log transformed PREDIM [mg kg^−1^ min^−1^]: 1.09±0.35 vs. 0.84±0.44, *p*=0.032; mean glucose [mg/dl]: 105±13 vs. 121±25, *p*=0.011). The median increase in triglyceride concentrations from first to second visit was 41 mg/dl (IQR: 12–79 mg/dl). A detailed analysis of the correlation between maternal triglycerides and glucose metabolism at the second visit suggested that parameters of glucose disposal were rather related to baseline triglycerides than to the change in triglyceride levels between the two visits (Table 2). Six cases of GDM occurred in this study. The association between development of GDM and triglycerides as well as other lipids parameters is provided in supplemental material (Table S1). Although the limited sample size has to be considered for interpreting these results, maternal triglycerides at early gestation were associated with development of GDM (OR: 1.16, 95%CI: 1.03–1.34, *p*=0.022 for an increase of 10 mg/dl), whereas this association was not observed for total cholesterol, HDL cholesterol and LDL cholesterol.

**Table 1:** Glucometabolic parameters of the study sample at early gestation. Patients with and without subtle hypertriglyceridemia (NTG vs. HTG) at 12+0–22+6 weeks of gestation.

	NTG	HTG	p-value
Age (years)	29.0±5.1	30.9±5.1	0.129
BMIPG (kg/m^2^)	22.2 (20.5-24.0)	23.5 (22.2-27.6)	0.035
BMIV1 (kg/m^2^)	24.2 (22.0-26.4)	25.5 (24.1-29.3)	0.042
Parity	0.0 (0.0-1.0)	0.0 (0.0-1.0)	0.620
GDM previous pregnancy	3 (7.5%)	2 (7.4%)	1.000
TG (mg/dl)	117.7±20.8	217.8±48.6	<0.001
TC (mg/dl)	214.0±39.8	231.6±40.1	0.082
LDL-C (mg/dl)	112.6±31.1	117.5±32.7	0.546
HDL-C (mg/dl)	80.5 (65.0-88.8)	66.0 (59.5-77.5)	0.067
G0 (mg/dl)	75.0 (72.0-78.0)	78.0 (73.0-82.0)	0.126
G-mean (mg/dl)	96.7 (84.3-105.3)	112.0 (96.8-131.3)	0.003
I0 (µU/ml)	6.17 (3.33-8.55)	7.25 (5.09-14.10)	0.068
I-mean (µU/ml)	33.0 (19.1-49.7)	54.3 (27.0-91.1)	0.090
CP0 (ng/ml)	1.50 (1.20-1.73)	1.70 (1.40-2.50)	0.040
CP-mean (ng/ml)	5.33 (4.22-6.63)	6.86 (4.90-8.92)	0.048
QUICKI (dimensionless)	0.16 (0.15-0.18)	0.16 (0.14-0.17)	0.074
ISI-comp (dimensionless)	8.51 (5.91-15.10)	6.13 (3.43-9.90)	0.045
PREDIM (mg kg^-1^ min^-1^)	3.63 (2.91-4.41)	2.67 (1.80-3.39)	0.006
AUC-I/AUC-G [0-120 min] (µU/mg)	41.5±24.9	50.6±31.6	0.318
BIS (pmol min^-1^ m^-2^)	70.5±23.3	87.0±34.4	0.035
TIS (nmol/m^-2^)	39.7±13.4	48.3±18.7	0.046
ISSI-2 (dimensionless)	2.93 (2.30-3.73)	2.58 (1.86-3.12)	0.037

Data are expressed as mean±standard deviation or median and interquartile range. Values are given for pregestational body mass index (BMIPG), body mass index at visit 1 (BMIV1), triglycerides (TG), total cholesterol (TC), LDL cholesterol (LDL-C), HDL cholesterol (HDL-C), glucose (G), insulin (I) and C-peptide (CP) at fasting and as mean values during the OGTT.

*QUICKI* quantitative insulin sensitivity check index; *ISI*-comp, insulin sensitivity composite index; *PREDIM* predicted M; overall insulin secretion (AUC-I/AUC-G [0–120 min]); *BIS* basal insulin secretion rate; *TIS* total insulin secretion rate from C-peptide; *ISSI*-2, oral disposition index

**Table 2:** Association of glucometabolic parameters at V2 (24–28 weeks of gestation) with triglyceride levels at V1 (baseline visit) and their change (V2 – V1)

V2	Association with baseline TG (V1)		Association with change of TG (V2-V1)	
	rho	*p*-value	rho	*p*-value
G0	0.19	N.S.	0.14	N.S.
G-mean	0.42	<0.001	0.03	N.S.
I0	0.21	N.S.	0.09	N.S.
I-mean	0.30	0.023	− 0.00	N.S.
CP0	0.55	<0.001	0.14	N.S.
CP-mean	0.43	<0.001	0.19	N.S.
QUICKI	− 0.21	N.S.	− 0.10	N.S.
ISI-comp	− 0.29	0.026	− 0.05	N.S.
PREDIM	− 0.41	0.001	− 0.08	N.S.
AUC-I/AUC-G	0.14	N.S.	− 0.05	N.S.
BIS	0.55	<0.001	0.09	N.S.
TIS	0.40	0.002	0.15	N.S.
ISSI-2	− 0.21	N.S.	− 0.03	N.S.

Data are expressed as Spearman’s correlation coefficient (rho). Values are given for glucose (G), insulin (I) and C-peptide (CP) at fasting and as mean values during the OGTT.

*QUICKI* quantitative insulin sensitivity check index; *ISI*-comp insulin sensitivity composite index; *PREDIM* predicted M; overall insulin secretion (AUC-I/AUC-G [0–120 min]); *BIS* basal insulin secretion rate; *TIS* total insulin secretion rate from C-peptide; ISSI-2, oral disposition index

## Discussion

This study aimed at investigating pathophysiological patterns of glucose metabolism in association with fasting triglyceride levels. We found that moderate hypertriglyceridemia at the start of pregnancy was related to impaired insulin action and *β*-cell function. This was accompanied by elevated glucose levels at mid-gestation. Consequently, increased serum triglycerides were associated with a higher risk for the development of GDM.

In the non-pregnant state, increased levels of triglycerides are a well-known characteristic of dyslipidemia associated with insulin resistance and are considered as an important risk factor for the development of diabetes and cardiovascular disease [17][18][19]. However, in the last decades, research interest in dyslipidemia in pregnancy was low, although more recently the spotlight shifted. It is well known that during course of physiologic gestation, plasma triglycerides concentrations increase due to impaired removal by lipolytic enzymes (such as lipoprotein lipase) [20]. This process may be further triggered by overweight or obesity, as a recent study published by Bozkurt *et al.* found that obesity was associated with abnormal lipid constellation and mainly elevated triglycerides concentration, maybe as a consequence of impaired insulin action in these patients [21]. This is in line with the results of our study, demonstrating decreased indices of insulin sensitivity in mothers with moderate hypertriglyceridemia even at early pregnancy, although we found that the association between increased triglycerides and impaired insulin action was independent of BMI, suggesting an association between maternal triglycerides and glucose metabolism which is independent of overweight or obesity status. Moreover, we found that parameters of glucose metabolism at the second visit were rather related to baseline triglycerides than to the change in triglyceride levels between the two visits.

Some studies have investigated the association between lipid levels and glucose metabolism in pregnancy: Sanchez-Vera *et al.* assessed differences in maternal lipid content in women who developed GDM as compared to normal glucose tolerant control women and observed elevated levels of triglycerides in women with GDM in the first trimester, already before GDM was diagnosed [22]. In another study, Bao *et al.* characterized longitudinal changes in lipid profiles throughout pregnancy and examined the association of plasma lipid levels with risk of GDM. The authors concluded that higher triglyceride levels in early and mid-pregnancy were significantly associated with greater risk of GDM manifestation [23]. Furthermore, Enquobahrie *et al.* examined maternal lipid levels in early pregnancy and the risk of GDM and observed that elevated triglycerides were positively associated with GDM [24]. Although the number of GDM cases is small in our study, what is a limitation of our work, the above-mentioned three studies are in accordance with our results, indicating that pregnant women with elevated triglycerides in early pregnancy are at increased risk for GDM. This association can be explained by a higher degree of insulin resistance, which is not adequately compensated by increased *β*-cell secretion. We observed not only impaired insulin sensitivity, but also lower ISSI-2 levels indicating insufficient compensation by the *β*-cells. Thus, elevated triglycerides at early gestation may be regarded as a potential biomarker for prediabetic disorders that will lead to hyperglycemia when pregnancy progresses and the physiologic environment changes. Although the establishment of early risk assessment tools is challenging due to the heterogeneity of the disease [25], a recent study by Benhalima *et al.* found that the inclusion of biomarkers such as triglycerides improves the accuracy of risk assessment models to predict the risk for the development of GDM at the start of pregnancy, as also underlined by the data of our study [26]. Of note, we observed no association between cholesterol (LDL, HDL or total cholesterol) with GDM development.

To our knowledge, this is the first study investigating the cross-link between elevated triglycerides and parameters of glucose metabolism, and we identified moderate hypertriglyceridemia as a potential risk factor for glycemic disorders in pregnancy. Although this aspect is novel, our study was not designed to assess implications for the newborns. This may be of additional interest, as maternal triglycerides are recognized as an important and physiologic fuel substrate for the growing fetus, but with a growing body of evidence proposing that increased maternal triglycerides correlate with excess fetal growth. Of note, maternal triglycerides have been associated with increased rates of large for gestational age newborns [27][28][29]. Moreover, associations with hypertension and preeclampsia were described indicating that further research is necessary to assess the potential role of elevated triglycerides as a risk factor for mothers and children [30].

Distinguishing independent contributions of pre-pregnancy obesity, pre-pregnancy insulin resistance or maternal lipid levels to impaired insulin resistance are challenging. We conclude that even moderately elevated triglycerides at the beginning of pregnancy are associated with impaired insulin action and β-cell dysfunction and hence with a potential risk factor for GDM development.

## Conclusion

This study investigated pathophysiological patterns of maternal glucose metabolism during pregnancy in association with fasting triglyceride levels. Hypertriglyceridemia at the start of pregnancy was closely related to impaired insulin action and β-cell function. Women with hypertriglyceridemia had higher mean glucose levels in early- and mid-gestation. Consequently, increased serum triglycerides were associated with a higher risk for the development of GDM.

## Electronic supplementary material

Below is the link to the electronic supplementary material.

Supplementary file1 (DOCX 12 kb)

## Data Availability

The datasets used and analyzed during the current study are available from the corresponding author on reasonable request.

## Abbreviations

BIS: Basal insulin secretion
BMI: Body mass index
CI: Confidence intervals
CP: C-peptide
GDM: Gestational diabetes mellitus
HTG: Moderate triglyceridemia
IQR: Interquartile range
NTG: Normal triglyceridemia
OGTT: Oral glucose tolerance test
OR: Odds ratio
QUICKI: Quantitative insulin sensitivity check index
TG: Triglycerides
TIS: Total insulin secretion
