# Supplementary material for: Association between maternal triglycerides and disturbed glucose metabolism in pregnancy
Source: Acta Diabetol. 2021 Jan 2;58(4):459–65. doi: 10.1007/s00592-020-01644-z (PMC8053660; doi:10.1007/s00592-020-01644-z)
Supplement: Supplementary file 1 — Supplementary file1 (DOCX 12 kb) [file 592_2020_1644_MOESM1_ESM.docx]

**Supplemental Material**

**Table S1:** Association of lipid parameters with GDM development

|  | **OR** | **95%CI** | **p-value** |
| --- | --- | --- | --- |
| Age (years) | 1.47 | 1.17 - 2.06 | 0.005 |
| BMIPG (kg/m²) | 1.34 | 1.15 - 1.68 | 0.001 |
| BMIV1 (kg/m²) | 1.38 | 1.64 - 1.74 | 0.001 |
| TG (mg/dl) × 10-2 | 1.16 | 1.03 - 1.34 | 0.022 |
| TC (mg/dl) | 1.00 | 0.98 - 1.02 | 0.971 |
| LDL-C (mg/dl) | 0.99 | 0.97 - 1.02 | 0.618 |
| HDL-C (mg/dl) | 0.98 | 0.93 - 1.02 | 0.391 |

Data are expressed as odds ratio (OR) and 95% confidence intervals (95%CI). Values are given for pregestational body mass index (BMIPG), body mass index at visit 1 (BMIV1), triglycerides (TG), total-cholesterol (TC), LDL-cholesterol (LDL-C), HDL-cholesterol (HDL-C);
